# Incidence and management of Lyme disease: a Scottish general practice retrospective study

**DOI:** 10.3399/BJGPO.2023.0241

**Published:** 2024-08-07

**Authors:** Sally Mavin, Swapna Guntupalli, Michael Robb

**Affiliations:** 1 Scottish Lyme Disease and Tick-borne Infections Reference Laboratory, Raigmore Hospital, Inverness, UK; 2 Public Health Directorate, NHS Highland, Inverness, UK

**Keywords:** Lyme disease, incidence, general practice

## Abstract

**Background:**

The true burden of Lyme disease in primary care in Scotland is unknown. Epidemiological data are currently based on laboratory-confirmed reports as there is no mandatory reporting of clinical cases.

**Aim:**

To analyse data from general practice in NHS Highland (North) over a 6-year period to assess the incidence and management of Lyme disease in primary care.

**Design & setting:**

This was a retrospective descriptive study. Study data from 2017 to 2022 were extracted from all 63 general practices within NHS Highland (North).

**Method:**

Consultations for Lyme disease were identified via Lyme-related clinical Read codes, requests for borrelia tests , free text, ‘tags’ and/or Lyme disease antibiotic scripts.

**Results:**

Using Read codes to identify patients with Lyme disease or suspected Lyme disease gave an estimated average annual incidence of 124/100 000 population, which was 2.1 times more than estimates based solely on laboratory-confirmed reports. The incidence figures increased 5.2 times (362/100 000 population) when patients with Lyme disease or suspected Lyme disease (identified via Read codes, laboratory test requests, and free text tags) who were given antibiotic treatment were taken into account. Local ‘hot spots’ of infection were identified. Analysis of the antibiotic data indicates that antibiotic prescribing in NHS Highland largely follows the National Institute for Health and Care Excellence (NICE) guidelines.

**Conclusion:**

This data analysis pathway can, and should, be rolled out across the whole of Scotland to assess the incidence and management of Lyme disease in primary care and allow appropriate allocation of resources.

## How this fits in

The true burden of Lyme disease in primary care in Scotland is unknown. This study indicates that the incidence may be more than five times higher than that suggested by current laboratory data. Better data recording will improve the accuracy of primary care incidence data. These findings will help target education and intervention measures and will help to quantify the financial impact on healthcare.

## Introduction

The true burden of Lyme disease in primary care in Scotland is unknown. Epidemiological data are currently based on laboratory-confirmed reports as there is no mandatory reporting of clinical cases.^1^ Laboratory testing is recommended for all patients with suspected Lyme disease, with the exception of those with an erythema migrans (EM) rash. These patients should be treated empirically without laboratory confirmation as tests are often negative with this early presentation, which is in itself clinically diagnostic.^2^ Thus, it is inevitable that a large proportion of cases of Lyme disease are not included in national incidence figures, as these figures are taken from laboratory data alone.

Data on laboratory-confirmed cases of Lyme disease from 2008 to 2013 estimated that the average incidence was 6.8 per 100 000 for Scotland, rising to 44.1 per 100 000 in NHS Highland.^3^ Although almost half of the laboratory-confirmed cases of Lyme disease in Scotland are from NHS Highland, this area only contains a population of 320 860 (5.9% of the national population), despite comprising 42% of the total land mass of Scotland.^4^ Studies using primary care data in England estimated that the real incidence could be 2.35–3 times higher than suggested by figures based on laboratory-confirmed cases.^5,6^ Understanding the burden of Lyme disease throughout Scotland is essential as it will help target education and intervention measures and will aid assessment of the financial impact on healthcare.

In 2018 the National Institute for Health and Care Excellence (NICE) published guidelines for the diagnosis and management of Lyme disease, NG95^2^, to ensure prompt and consistent diagnosis and treatment. There is no mandate to follow NICE guidelines in Scotland but it is assumed that the guidelines have been universally adopted in both primary and secondary care, although to date no studies have assessed this.

The aim of this study was to analyse data from general practices in NHS Highland (North) over a 6-year period to assess the incidence and management of patients treated for Lyme disease in primary care.

## Method

### Patient data

Data routinely collected from all 63 general practices in NHS Highland (North), covering a population of 235,430^7^, from January 2017 to December 2022 were extracted using EScro (Enhanced Service contract reporting options).^8^

In primary care, patient data are recorded in electronic records via the use of Read codes and free text. Read codes are used to record specific clinical features, symptoms, or diseases and should theoretically provide a quick and accurate means to analyse data. All patient encounters that had been assigned Lyme-related Read codes (Lyme disease or suspected Lyme disease and their associated symptoms) were identified and downloaded (Table 1).^6^

**Table 1.** Number of patients and encounters within NHS Highland (North) primary care assigned Lyme-related Read codes or identified by Lyme Read code, borrelia test request, or text tag and/or associated antibiotic, 2017–2022

Read code	Description	2017	2018	2019	2020	2021	2022	Total
AA41	EM	49	79	126	74	159	271	758 (37.4%)
1JN2	Suspected EM	47	82	143	72	128	92	564 (27.8%)
A8710	Lyme disease	65	70	90	65	86	80	456 (22.5%)
1JN1	Suspected Lyme disease	0	0	75	31	51	50	207 (10.2%)
A8711	Lyme neuroborreliosis	9	6	5	9	4	5	38(1.9%)
N010A	Arthritis in Lyme disease	0	0	0	1	0	2	3(0.1%)
M21y0	ACA	0	0	1	0	0	1	2(0.1%)
A8712	Lyme carditis	0	0	0	0	0	0	0
A8713	Borrelia lymphocytoma	0	0	0	0	0	0	0
Patient encounters assigned Lyme-related Read codes, *n*		170	237	440	252	428	501	2028
Patients assigned Lyme-related Read codes^a^, *n*		142	210	373	220	369	442	1756
Patients identified by Lyme Read code, borrelia test request or text tag and treated with antibiotics^b^, *n*		NA	NA	758	749	948	957	3412
Laboratory-confirmed cases of Lyme disease, *n*		66	112	126	126	190	210	830

^a^Patients may be assigned multiple Read codes per consultation or during subsequent visits. Therefore, the incidence data analysed were restricted to the number of unique patients with any of the Lyme or suspected Lyme Read codes assigned.

^b^Antibiotic data not available for 2017 and 2018.

ACA = acrodermatitis chronica atrophicans. EM = erythema migrans. NA = data not available.

To identify cases of Lyme disease or suspected Lyme disease that may not have been assigned Lyme-related Read codes, any patient encounters associated with a borrelia test request were also identified and downloaded. In addition, patient encounters associated with other less specific Read codes for Lyme disease (such as rash, chronic fatigue syndrome, or Bell’s palsy) were identified and downloaded if they were associated with text tags and/or certain antibiotic scripts. The text tags included any free text that included the terms: tick bite; erythema; migrans; burgdorferi; engorged tick; Lyme. The antibiotic scripts included those commonly used to treat Lyme disease (supplementary Table S1).

Data downloaded for each patient encounter identified included patient Community Health Index (CHI) number,^9^ encounter ID, practice code, sex, age band, date, Read code and descriptor, text tag, and associated antibiotic data (drug name, dose, and quantity). Records that did not include this CHI number were excluded.

### Laboratory data

Data were obtained from samples sent to the Scottish Lyme Disease and Tick-borne Infections Reference Laboratory (SLDTRL) from NHS Highland (North) patients in primary care from 2017 to 2022. The number of laboratory-confirmed cases was taken as the number of patients that produced a positive *Borrelia burgdorferi* immunoglobulin G (IgG) and/or immunoglobulin M (IgM) immunoblot for the first time (with the exclusion of patients with an isolated IgM positive result with documented onset >10 weeks).

### Analysis

The number of cases of Lyme disease and suspected Lyme disease, based on the number of patients assigned a Lyme-related Read code, as well as the number of patients identified via Lyme-related Read codes, borrelia test requests, and text tags who were prescribed antibiotics commonly used to treat Lyme disease, were compared with the number of laboratory-confirmed cases of Lyme disease during the 6-year study period. Estimated annual incidence per NHS Highland (North) population was determined.^7^ Antibiotic prescribing patterns (drug, duration, number of courses) were then examined.

The average annual incidence per 10 000 practice population was plotted on a map of NHS Highland (North) using natural breaks (Jenks) distribution which clusters data into groups that minimise the within-group variance and maximise the between-group variance. Representative case numbers were simultaneously plotted. The map was produced with ESRI ArcGIS Pro (3.1.2) software and Ordnance Survey base mapping.

## Results

In total, 1756 unique patients within primary care in NHS Highland (North) were assigned a Lyme-related Read code between January 2017 and December 2022 (Table 1). If the assignation of Lyme-related Read codes was used to identify cases of Lyme disease this would equate to an estimated average annual incidence of 124/100 000 population. This compares with 830 laboratory-confirmed cases over the same period, an estimated average annual incidence of 59/100 000 population (multiplication factor of 2.1) (Table 1).

The EM Read code was the most common (37.4%), followed by suspected EM (27.8%), Lyme disease (22.5%), then suspected Lyme disease (10.2%), and Lyme neuroborreliosis (1.9%).

In total, 3412 patients with Lyme disease or suspected Lyme disease, identified by Lyme-related Read codes, borrelia test requests, and text tags, were treated with antibiotics from January 2019 to December 2022 (Table 1). This would equate to an estimated average annual incidence of 362/100 000 population for NHS Highland (North), which is significantly higher than the number of patients assigned Lyme-related Read codes for the same period (*n*=1404; incidence of 149/100 000), as well as the number of laboratory-confirmed cases for the same period (*n*=652; incidence of 69/100 000) (multiplication factor of 5.2).

**Figure 1.**
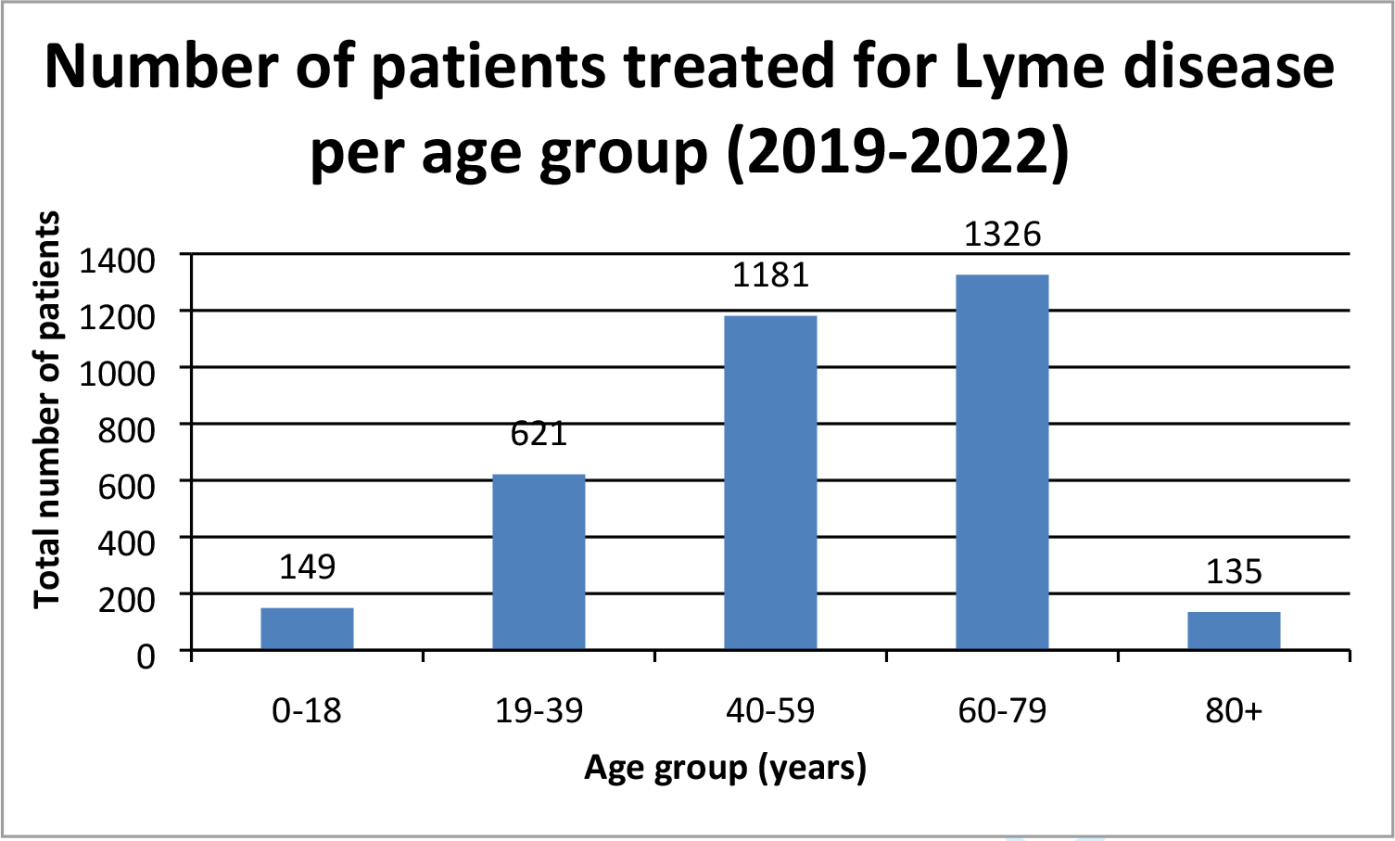
Age distribution of patients treated for Lyme disease, identified by Lyme Read code, borrelia test request, or text tag (2019-2022,*n* = 3412). Antibiotic data not available for 2017 and 2018.

When the demographics of these 3412 patients were analysed, there was a similar number of females and males (51 versus 49%), and the highest proportion of patients (38.86%) was in the 60–79 years age group (Figure 1).

**Figure 2.**
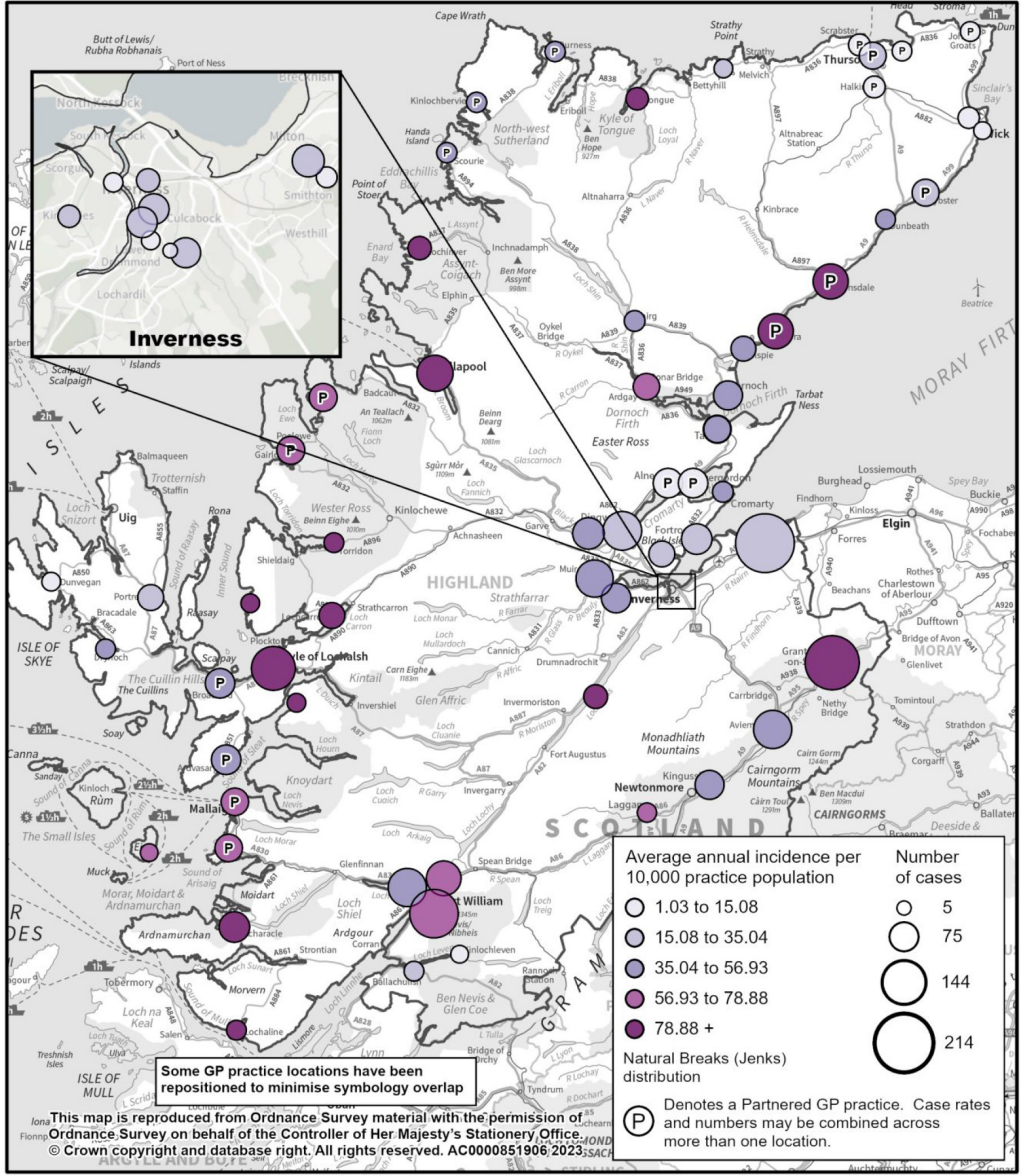
Number of patients treated for Lyme disease (cases) and average annual incidence per 10 000 practice population for general practices within NHS Highland (North)(2019–2022). Antibiotic data not available for 2017 and 2018.

The largest number of patients treated for Lyme disease in NHS Highland (North) were from Nairn (*n*=214), followed by Grantown-on-Spey (*n* = 190), Tweeddale (*n* = 162), Kyle (*n* = 137), and Dingwall (*n* = 111) medical practices (Figure 2). However, when the average annual incidence per 10 000 was examined for each practice (10 000 used as practice populations are low), the highest incidences were found in Tongue (127.1/10 000), Kyle (125.4/10 000), Acharacle (122.6/10 000), and Lochcarron (119.9/10 000) (Figure 2). Five medical practices (Brora and Helmsdale, Ullapool, Kyle, Tweeddale, and Grantown-on-Spey) had both a high number of cases (*n*>75) and a high incidence (>78.89 per 10 000).

There were 3519 courses of antibiotics prescribed to patients with Lyme disease or suspected Lyme disease over the 4-year period that antibiotic data was available (Table S1). As might be expected, 3376 (95.9%) scripts were for doxycycline 100 mg. In total, *n* = 189/3376 (5.60%) of these scripts were for 7 days, *n* = 739/3376 (21.89%) for 14 days, *n* = 2101/3376 (62.23%) for 21 days, and *n* = 85/3376 (2.52%) for 28 days. The remaining 262 were for other durations.

When the number of prescriptions per patient were examined, *n* = 3036/3261 (93.10%) patients received only one script and *n* = 211/3261 (6.47%) received two. Only 14 patients (0.43%) received three scripts and two patients (0.06%) received four.

## Discussion

### Summary

Although the incidence of Lyme disease in Scotland is low compared with that in many other European countries, the Highlands of Scotland have long been considered a hot spot, with estimated incidence figures comparable with some European countries where it is considered endemic.^3^ Analysis of Lyme-related Read codes shows that the incidence of Lyme disease within NHS Highland (North) may be 2.1 times higher than laboratory data indicate. However, this study suggests that the use of Read codes varies and that these alone are not sufficient to identify patients with Lyme disease or suspected Lyme disease, so referral to free text and antibiotic scripts is necessary. This is reflected in the fact that the incidence figures increased 5.2 times compared with laboratory data alone when all patients treated for Lyme disease (including those identified via free text tags) were taken into account.

As may be expected, 10 of the 15 practices with the highest number of cases (*n*>75) had a practice population >5000 (that is, in the top third), whereas nine of the 14 practices with the highest incidence ( >78.89/10 000) had a practice population of less than 1500. Five medical practices had both a high number of cases (*n*>75) and a high incidence (>788.9 per 100 000), perhaps reflecting the highest risk areas. These were Brora and Helmsdale, Ullapool, Kyle, Tweeddale, and Grantown-on-Spey. However, the data are based on where a patient is treated and not where they acquired the infection.

### Strengths and limitations

This is the first comprehensive study of Lyme disease data in primary care in Scotland, which has provided valuable information on the burden of the disease across a specific health board. Including cases of treated and suspected Lyme disease to estimate incidence as well as those that are clinically confirmed may potentially lead to an overestimation. Many symptoms of Lyme disease mimic those of other diseases or conditions, so the NICE guidelines recommend laboratory testing for all cases of suspected Lyme disease except those presenting with EM and state that treatment should be considered while waiting for test results if there is a high clinical suspicion.^2^ Thus, it is possible that some suspected cases may not have had Lyme disease. Conversely, using suspected Read codes or free text may simply reflect a reluctance to assign the more definitive EM or Lyme disease Read codes, and case numbers could potentially be underestimated if these are not included. There is often a misconception that EM should present as the textbook target or bullseye rash with central clearing. The appearance of EM can vary considerably, thus European case definitions define EM as a spreading rash (>5 cm) from the site of a tick bite, with or without central clearing.^10^ Regardless, inclusion of both suspected and clinically confirmed cases is important when assessing disease burden.

Data studies rely on the quality of the data available. Unfortunately, some individual practice data were missing. There were obvious omissions in the data for 2017–2019 as there were more borrelia test requests from NHS Highland patients than the number of encounters associated with Lyme disease identified from patient records. This may reflect a slow uptake of the use of Lyme-related Read codes, which were only introduced in 2014. It could also, in part, be a result of a lack of antibiotic data available for 2017 and 2018 (because of the amount of data that can be handled by the data retrieval system). In addition, it is likely that there were issues or omissions with the input of borrelia test results, which were also used to identify Lyme patients. During 2017–2019, borrelia test results were manually entered into GP patient records. Test results were only automatically incorporated from January 2021. Indeed, there were more GP encounters related to Lyme disease identified than samples tested for borrelia from 2020–2022, which may reflect an increase in the quality of the data.

### Comparison with existing literature

The study by Tulloch *et al* ^6^ analysed the same Lyme-related Read codes from population-based primary care data from the Health Improvement Network (THIN), which collects data from general practices using the VISION practice management software (9% of all GP practices in England). Their estimated incidence of Lyme disease in Scotland was extrapolated to be 10.3/100 000 for 2008–2013. This was 1.5 times higher than the incidence figure of 6.8 per 100 000 for Scotland calculated from laboratory-confirmed cases for the same period.^3,6^ Interestingly, when using a similar approach to the current study (analysing Read codes, free text and antibiotic scripts) but analysing data from the Clinical Practice Research Datalink (CPRD), which covers approximately 8% of the UK population, Cairns *et al* ^5^ extrapolated the estimated incidence in Scotland to be 37.3/100 000 for 2010–12, which was 5.5 times higher than the laboratory data-derived incidence figure of 6.8 per 100 000 for Scotland for 2008–2013^5^. Both of these results were similar to our findings.

The finding that 65.2% of patients with Lyme-associated Read codes were assigned EM or suspected EM Read codes was much higher than the 27.9% reported by Tulloch *et al* ^6^ when analysing UK primary care data, which may reflect more awareness and confidence in assigning the EM or suspected EM Read codes in this area of Scotland, which has a high incidence of Lyme disease. The slight female preponderance was similar to that seen by Cairns *et al* ^5^ and Tulloch *et al*.^6^ Likewise, the peak in cases in the 60–79 years age group was similar to that of Tulloch *et al* ^6^ and only slightly higher than Cairns *et al.* ^5^ Interestingly, there continues to be a lower proportion of cases in people ages <20 years in Scotland than in England (4.4 versus 13.7%).^5^

Analysis of the antibiotic data indicates that antibiotic prescribing in NHS Highland largely follows NICE guidelines: doxycycline 100 mg (twice daily) for 21 days (28 days for patients with Lyme arthritis or acrodermatitis chronic atrophicans).^2^ The NICE guidelines proposed a treatment duration of 21 days for the majority of cases of Lyme disease to standardise treatment and remove the ambiguity of previous guidance, which advocated 14–21 days of treatment. Shorter courses (7 and 14 days) were prescribed to 27% of patients. While this is not compliant with the NICE guidelines, the Infectious Diseases Society of America (IDSA) 2020 guidelines recommend that patients with EM are treated with 10 days of doxycycline.^11^ Likewise, a recent study supports 7 days of doxycycline for European patients with solitary EM.^12^

NICE guidelines suggest a second course with an alternative antibiotic for patients with ongoing symptoms (such as refractory arthritis) if treatment may have failed, but further treatment courses are not recommended, as persistent symptoms following treatment (post-treatment Lyme disease syndrome) are not thought to be caused by persisting infection.^2^ Only 16 patients were prescribed three or more courses of antibiotics associated with a Lyme-related episode. Although re-infection cannot be completely ruled out it appears that these patients had persisting symptoms, mainly of chronic fatigue, and highlights potential overtreatment and non-compliance with the guidelines.

### Implications for research and practice

Using Read codes to identify patients with Lyme disease indicated that current incidence figures in primary care within NHS Highland are 2.1 times higher than those based solely on laboratory-confirmed reports. However, when the number of patients actually treated for Lyme disease was taken into account the incidence figures could be 5.2 times higher than suggested by data from laboratory-confirmed cases. Analysis of the antibiotic data indicates that antibiotic prescribing in NHS Highland largely follows NICE guidelines.

This GP data analysis pathway can, and should, be rolled out to assess the incidence and management of Lyme disease in primary care throughout the whole of Scotland to allow appropriate resources to be allocated.
